# Direct and indirect effects of estrogens, androgens and intestinal microbiota on colorectal cancer

**DOI:** 10.3389/fcimb.2024.1458033

**Published:** 2024-11-26

**Authors:** Zihong Wu, Yi Sun, Wenbo Huang, Zhenzhen Jin, Fengming You, Xueke Li, Chong Xiao

**Affiliations:** ^1^ Traditional Chinese Medicine (TCM) Regulating Metabolic Diseases Key Laboratory of Sichuan Province, Hospital of Chengdu University of Traditional Chinese Medicine, Chengdu, China; ^2^ Institute of Oncology, Chengdu University of Traditional Chinese Medicine, Chengdu, China; ^3^ Oncology Teaching and Research Department, Chengdu University of Traditional Chinese Medicine, Chengdu, China

**Keywords:** colorectal cancer, estrogens, androgens, intestinal microbiota, direct and indirect effects

## Abstract

Sex differences in colorectal cancer (CRC) has received considerable research attention recently, particularly regarding the influence of sex hormones and the intestinal microbiota. Estrogen, at the genetic and epigenetic levels, directly inhibits CRC cell proliferation by enhancing DNA mismatch repair, regulating miRNAs, blocking the cell cycle, and modulating ion channels. However, estradiol’s activation of GPER promotes oncogene expression. Conversely, androgen contributes to epigenetic dysregulation and CRC progression via nuclear receptors while inducing apoptosis through membrane receptors. Specific gut microorganisms produce genotoxins and oncogenic metabolites that damage colonic cell DNA and contribute to cancer induction. Regarding the tumor microenvironment, estrogen mitigates intestinal inflammation, reverses immunosuppression, increases gut microbiome diversity and commensal bacteria abundance, and decreases pathogen enrichment. On the contrary, androgen disrupts intestinal microecology, diminish immunotherapy efficacy, and exacerbate colonic inflammation and tumor growth. The impact of estrogen and androgen is closely tied to their receptor status, elucidating their dual roles in CRC pathogenesis. This review comprehensively discusses the direct and indirect effects of sex hormones and the intestinal microbiota on CRC, considering environmental factors such as diet and lifestyle to propose novel prevention and treatment strategies.

## Graphical Abstract

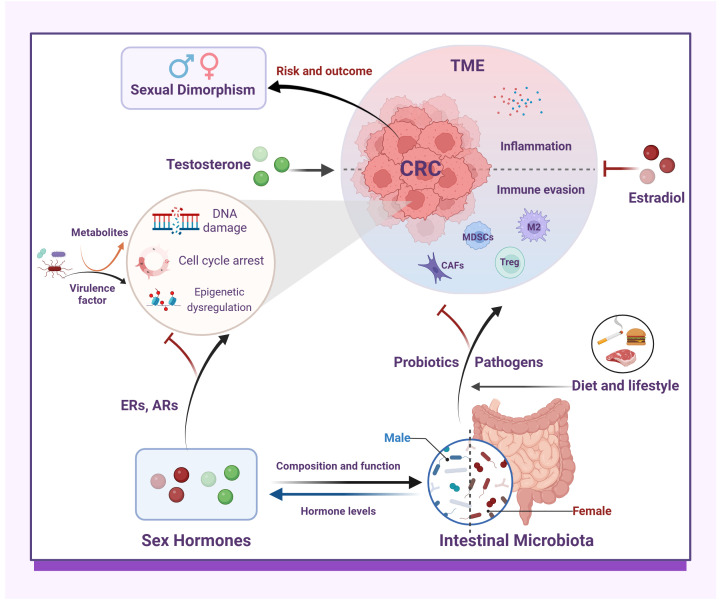

## 1 Introduction

Colorectal cancer (CRC), a common malignant tumor of the digestive system, has exhibited an upward trend in morbidity and mortality in recent years (Siegel et al., 2024). Men typically display a higher incidence rate and poorer prognosis (Rubin et al., 2020; Baraibar et al., 2023). In the comprehensive investigation of CRC pathogenesis and prevention strategies, sex differences have emerged as a significant factor. Growing evidence indicates that variations in sex hormone levels, particularly estrogen and androgen, along with the intestinal microbiota, play a crucial role in the sexual dimorphism of CRC (Abancens et al., 2020; Salvador et al., 2023; Chang et al., 2024). Estrogen, for instance, may exert an inhibitory effect on CRC development in certain scenarios, whereas testosterone could stimulate the proliferation and metastasis of CRC cells (Barzi et al., 2013; Haziman et al., 2019; Song et al., 2021a). Furthermore, sex hormones may indirectly modulate colorectal carcinogenesis by influencing the intestinal microenvironment (Jiang et al., 2021; Matarrese et al., 2021). However, the levels of sex hormones are influenced by numerous factors such as genetic background, lifestyle choices, and environmental conditions, thereby increasing the complexity of CRC pathogenesis (Zhang et al., 2019). Consequently, the role of sex hormones in the development of CRC remains controversial. the intestinal microbiota, a vast and intricate ecosystem within the human body, significantly impacts host health. Recent findings highlight a strong link between the intestinal microbiota and sex disparities in CRC (Lin et al., 2019; Koyande et al., 2022; Yang et al., 2023). On one hand, there are notable differences in the composition and function of the intestinal microbiota between males and females, potentially affecting immune function and metabolic processes of the gut mucosa, consequently influencing CRC risk (Fan et al., 2021). On the other hand, the intestinal microbiota can impact the host’s sensitivity and resistance to chemotherapeutic agents, which may contribute to sex-specific variations in CRC treatment outcomes and prognosis (Lin et al., 2019). Importantly, various factors like sex hormone levels, dietary habits, and lifestyle choices can influence sex differences in the intestinal microbiota (Song et al., 2020c). Therefore, the relationship between the intestinal microbiota and sex disparities in CRC likely involves multiple complex interactions.

The recent exploration of concepts like the micro-genderome and the sex hormone-gut microbiome axis has highlighted the intricate interplay between sex hormones and the gut microbiota (Wallis et al., 2017; Ma and Li, 2019; Vemuri et al., 2019; Calcaterra et al., 2022). However, the precise mechanisms governing the interactions among CRC sexual dimorphism, sex hormone signaling, and the intestinal microbiota remain incompletely understood. This review delves into the bidirectional relationship between sex hormone signaling and the intestinal microbiota, with a focus on the direct effects (genetic and epigenetic alterations) and indirect pathways (modulation of inflammation and immune microenvironment) of sex hormones and the intestinal microbiota on CRC. Additionally, non-biological sex differences (such as diet and lifestyle) in the intestinal microbiota in CRC will be briefly discussed for comprehensive understanding. This comprehensive analysis is important for deciphering the pathogenesis of CRC and devising targeted prevention and treatment approaches.

## 2 Bidirectional communication between sex hormones and the intestinal microbiota

### 2.1 Sex hormones influence the intestinal microbiota composition and diversity

Sex differences in the gut microbiota composition have been observed in both clinical samples and animal models. Research has shown that females generally exhibit higher microbial α-diversity and a higher *Firmicutes*/*Bacteroidetes* (F/B) ratio (Hokanson et al., 2024). However, the higher microbial α-diversity in women may be attributed to their relatively lower *Bacteroidetes* abundance, as evidence suggests a negative association between *Bacteroidetes* abundance and α-diversity (Thackray, 2019; Zhang et al., 2022). Factors like geographic location can also impact the F/B ratio (Manor et al., 2020). While summarizing sex-based differences in bacterial taxa abundance is challenging due to inconsistent findings across studies, it can be inferred that estradiol and testosterone play a role in maintaining gut microbiota diversity. Certain bacteria have been linked to sex hormones, with men having high serum testosterone levels showing enrichment for specific genera like *Ruminococcus*, *Prevotella*, *Fusobacterium*, *Dorea*, *Acinetobacter*, and *Megamonas*, while women with higher serum estradiol levels exhibit positive correlations with *Bacteroidetes* phyla, *Akkermansia* and *Ruminococcus* (del Castillo-Izquierdo et al., 2022). Additionally, the use of hormonal contraceptives has been found to alter gut microbiota composition in women (Sinha et al., 2019). For example, Oral contraceptives are associated with an increased abundance of species such as *Bacteroides caccae*, *Coprobacillus unclassified*, and *Rothia mucilaginosa* (Sinha et al., 2019). *Bacteroides* represent a significant group of microorganisms in the intestine (Magne et al., 2020), while *Rothia mucilaginosa* has been closely linked to the development of Crohn’s disease (Gevers et al., 2014). Previous studies, along with recent epidemiological evidence, have consistently indicated that the use of antiandrogen oral contraceptives reduces the risk of CRC, suggesting a potential protective role for estrogen (Bosetti et al., 2009; Bennink et al., 2024). However, there remains a lack of comprehensive research regarding the relationship between the duration of oral contraceptive use and the risk of side effects, highlighting the need for more high-quality evidence to support its availability. Animal studies have further explored the bidirectional relationship between sex hormones and gut microbiota, revealing significant differences in microbial populations between male and female mice. For instance, certain genera like *Allobaculum*, *Anaeroplasma*, and *Erwinia* were more abundant in males, while females had higher levels of *Ruminococcus*, *Dorea*, and *Coprococcus* (Org et al., 2016). Gonadectomy results in dysbiosis of the microflora (Org et al., 2016; del Castillo-Izquierdo et al., 2022; Song et al., 2023b; Cross et al., 2024). Specifically, in mice fed high-fat and high-sugar diets, ovariectomized (OVX) mice showed decreased abundance of *Akkermansia*, while orchiectomized (ORX) mice exhibited increased abundance of the *Ruminococcacea* family (Org et al., 2016). Microbial diversity and F/B ratios were notably reduced in ORX_mice treated with azoxymethane/dextran sodium sulfate (AOM/DSS) (Song et al., 2023b). The alterations in microbiota composition and diversity were significantly reversed with estrogen or testosterone replacement (Org et al., 2016; Song et al., 2023b). Furthermore, ovariectomy also led to increased fecal β-glucuronidase activity (Cross et al., 2024). The apparently contradictory findings on gut microbiome composition between humans and mice suggest that genetic variability among strains and environmental factors (such as diet and exposure to carcinogens) may obscure sex differences.

**Figure 1:**
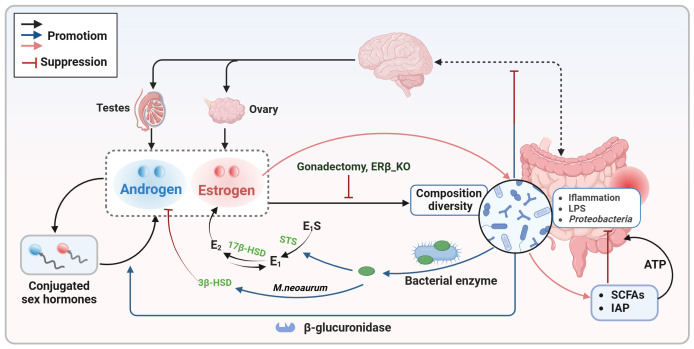
Bidirectional communication between sex hormones and the intestinal microbiota. Sex hormones (estrogen and androgen) play a crucial role in shaping the composition and diversity of the intestinal microbiota. Estrogen specifically supports the growth of SCFAs-producing bacteria and increases the expression of antimicrobial peptides to decrease the presence of pathogens. Conversely, the gut microbiota also influences sex hormone levels by expressing enzymes like β-glucuronidase and certain bacterial enzymes, as well as through interactions with the gut-brain axis. E1, estrone; E2, estradiol; E1S, estrone sulfate; E2S, estradiol sulfate; HSD, hydroxysteroid dehydrogenase; IAP, intestinal alkaline phosphatase; LPS, lipopolysaccharide; SCFAs, short chain fatty acids; STS, steroid sulfatase.

Estrogen is a key regulator of the gut microbiota, promoting the growth of bacteria that produce short-chain fatty acids (SCFAs). These SCFAs, such as butyric acid, are important metabolites resulting from gut microbe fermentation, crucial for maintaining intestinal homeostasis (Sun et al., 2018). Once produced in the colon, SCFAs are quickly absorbed by colonic cells and enter the citric acid cycle in mitochondria to generate ATP, providing energy to the cells (Koh et al., 2016; Dalile et al., 2019). In addition, SCFAs have anti-inflammatory properties, stimulate reactive oxygen species production, improve intestinal barrier function, modulate immune responses, and exhibit anti-CRC activity (Parada Venegas et al., 2019; Huang et al., 2023). Intestinal alkaline phosphatase (IAP), an antimicrobial peptide regulated by gut microbes, is up-regulated by estrogen, reducing Proteobacteria abundance and lipopolysaccharide biosynthesis to prevent chronic enteritis (Kaliannan et al., 2018). These findings suggest that sex hormone signaling can influence gut microbiota composition and diversity (**Figure 1**). However, studies on sex-related differences in the gut microbiota have yielded inconsistent results due to variations in study populations, methods, geography, and diet, posing a challenge in the field of gut microbiome research.

### 2.2 The intestinal microbiota involves in sex hormone metabolism

Not only sex hormone signaling alters the intestinal microbiota composition and diversify, but also the latter participate in the circulation and degradation of sex hormones (**Figure 1**). Certain bacteria, particularly in the gut microbiota of premenopausal women, possess genes involved in both the biosynthesis and degradation of sex hormones. These gut bacteria have the ability to metabolize sex hormones and their precursors (del Castillo-Izquierdo et al., 2022). Gut microbial β-glucuronidase is a key enzyme responsible for converting conjugated estrogens and androgens into their active forms (Hu et al., 2023). Through the action of β-glucuronidase, biologically active free sex hormones are reabsorbed via the enterohepatic circulation, thereby maintaining the body’s sex hormone levels and downstream physiological effects (Hu et al., 2023). Imbalances in the intestinal microbiota can disrupt the activity of β-glucuronidase, leading to a disruption in the breakdown of conjugated sex hormones and a decrease in free sex hormone levels, potentially resulting in various diseases (Hu et al., 2023). A study examining the impact of gut microbiota on intestinal androgen metabolism compared the levels of testosterone and dihydrotestosterone in the distal intestines of mice with normal gut microbiota and germ-free mice (Collden et al., 2019). The results revealed significantly higher levels of free dihydrotestosterone in the distal gut and feces of young adult males. In contrast, germ-free mice exhibited elevated levels of glucuronidated testosterone and dihydrotestosterone but lower levels of free dihydrotestosterone in the distal intestine (Collden et al., 2019). In addition, changes in intestinal flora appear to trigger androgen deficiency, causing metabolic disorders that lead to early death (Harada et al., 2020). These findings suggest a link between free estrogen and testosterone levels in the blood or gut and the presence of gut microbiota capable of metabolizing sex steroid hormones, implying that clinical testing of the abundance of specific bacteria in the feces may be able to reflect serum sex hormone levels.

The synthesis and metabolism of sex hormones involve a complex series of enzymatic reactions. Recent evidence suggests that specific bacterial enzymes, such as steroid sulfatase (STS), hydroxysteroid dehydrogenase (HSD), and steroid-17,20-desmolase, play a role in sex hormone biosynthesis alongside β-glucuronidase (Kwa et al., 2016; Thériault and Lin, 2019; Anbar et al., 2021). The conversion of estrone sulfate (E_1_S) and estradiol sulfate (E_2_S) to free estrone and estradiol is facilitated by STS. HSD17B_1_, HSD17B_7_, and HSD17B_12_ can convert estrone to estradiol, while HSD17B_2_ can metabolize estradiol back to estrone (Thomas and Potter, 2013). These findings indicate that the intestinal microbiota may impact estrogen bioavailability. Testosterone deficiency has been linked to depression-like behavior (Hauger et al., 2022). Studies have shown that *Mycobacterium neoaurum*, isolated from the feces of depressed patients with testosterone deficiency, can degrade testosterone *in vitro* (Li et al., 2022). *In vivo* experiments in rats confirmed that *M.neoaurum* reduced brain and serum testosterone levels, leading to depressive behavior, with 3β-HSD identified as the key enzyme responsible for degrading testosterone (Li et al., 2022). Moreover, certain bacteria in human feces have been found to metabolize estrogens and androgens (Rizzetto et al., 2018). While these studies support the role of bacterial enzymes in sex hormone metabolism, further research is needed to fully understand the complete bacterial pathways involved and their impact on human physiology. Beyond enzyme expression, gut microbes can influence sex hormone levels by directly affecting gonadal function (Shobeiri et al., 2022). Some gut microbes may interfere with endogenous sex hormone biosynthesis by modulating the hypothalamic-pituitary-gonadal axis *via* the gut-brain axis (So and Savidge, 2021; Hao et al., 2022; Hokanson et al., 2024).

Recent studies have indicated that the gut microbiota may influence the absorption of (phyto)estrogens, which in turn affects the ability of estrogens to bind to ERβ in the intestinal mucosa and counteract the activity of ERα, thereby playing a beneficial role in the prevention of colorectal neoproliferative lesions (Kumari et al., 2024). Consistent with this, estrogen primarily affects colonocytes through ERβ; notably, ERβ knockout in female mice results in decreased gut microbiota diversity and an increase in immune-damaging bacteria (Ibrahim et al., 2019; Ma et al., 2022). The gradual loss of ERβ during CRC development exacerbates the decrease in gut microbiota diversity and the enrichment of pathogens, such as the *Bacteroidetes* genus *Prevotellaceae* (Ibrahim et al., 2019). These findings suggest that the gut microbiota serves as a regulator of estrogen status, and that ERβ may inhibit the progression of colitis and colon cancer by modulating the gut microbiota. The human gut microbiota regulates estrogen metabolism primarily through the ‘estrogenome’—a collection of bacterial genes that encode enzymes such as β-glucuronidase, which activate estrogen and influence circulating levels. However, there is currently no evidence to suggest that changes in the gut microbiota affect the expression of ERα or ERβ.

Overall, sex hormones regulate the intestinal microbiota composition and diversity, which in turn can influence sex hormones levels (**Figure 1**; **Box 1**). This bidirectional communication is crucial in understanding sex differences in CRC. The following sections will delve into the direct effects and indirect pathways through which the sex hormone-gut microbiome axis affects the progression of CRC.

Box 1 **Bidirectional communication between sex hormones and the intestinal microbiota.**

**Table:** 

**Sex hormones influence the gut microbiota composition** • Sex differences in the intestinal microbiota composition have been observed in both humans and mice. • Estradiol and testosterone play a role in maintaining gut microbiota diversity.	**Gut microbial 6-glucuronidase** • β-glucuronidase is responsible for converting conjugated sex hormones into their active forms. • Intestinal microecological dysbiosis disrupts β- glucuronidase activity and reduces free sex hormone levels.
**Gonadectomy leads to intestinal microecological dysbiosis** • Ovariectomized mice shows decreased *Akkermansia* abundance, while orchiectomized mice exhibits increased *Ruminococcacea* abundance. • Ovariectomy leads to increased fecal β-glucuronidase activity.	**Specific bacterial enzymes** • STS, HSD, and steroid-17,20-desmolase regulate sex hormone biosynthesis. • The intestinal microbiota impact estrogen bioavailability. • 3β-HSD produced by *Mycobacterium neocurum* can degrade testosterone.
**Estrogen is a key regulator of the intestinal microbiota** • Estrogen promotes the growth of bacteria that produce SCFAs. • Estrogen upregulates the antimicrobial peptide IAP to reduce *Proteobacteria* abundance and lipopolysaccharide biosynthesis. • The gradual loss of ERβ during CRC development exacerbates the decrease in gut microbiota diversity and pathogens enrichment	**Gonadal function** • Certain gut microbes interfere with sex hormone biosynthesis by modulating the hypothalamic-pituitary- gonadal axis *via* the gut-brain axis.

CRC, colorectal cancer; ERβ, estrogen receptor-beta; HSD, hydroxysteroid dehydrogenase; IAP, intestinal alkaline phosphatase; SCFAs, short chain fatty acids; STS, steroid sulfatase.

## 3 Direct effects of sex hormones and the intestinal microbiota on CRC: genetic and epigenetic alterations

### 3.1 Sex hormone-related genomic and non-genomic signals

In the classical pathway, sex steroid hormones bind to nuclear receptors in the cytoplasm, forming ligand-receptor complexes that induce conformational changes in the receptor, leading to its translocation to the nucleus. There, the receptor interacts with specific DNA sequences to selectively regulate gene transcription. In addition to these classical genomic mechanisms, sex steroid hormones can activate signal transduction pathways through non-genomic signaling mechanisms that function independently of their nuclear role (Wilkenfeld et al., 2018). Non-genomic signaling typically occurs on a much faster time scale and is often referred to as fast signaling. Rapid signals generally involve the translocation of ribonucleic acids to the plasma membrane, where they can directly or indirectly activate kinase pathways, resulting in a variety of physiological effects (Simoncini and Genazzani, 2003; Norman et al., 2004; Wilkenfeld et al., 2018). It is crucial to emphasize that non-genomic pathways can also lead to genomic effects. Consequently, many reported genomic effects mediated by sex steroid hormones may actually be partially the indirect result of rapid non-genomic signaling. The complex and diverse structure-function relationships between sex steroid hormones and their nuclear and membrane-associated receptors, along with the capacity of the same receptor to elicit both genomic and rapid responses, complicate the understanding of the role of sex steroid receptors in CRC (Norman et al., 2004).

#### 3.1.1 Estrogen

##### 3.1.1.1 Mismatch repair, miRNAs, cell cycle and ion channels

**Figure 2:**
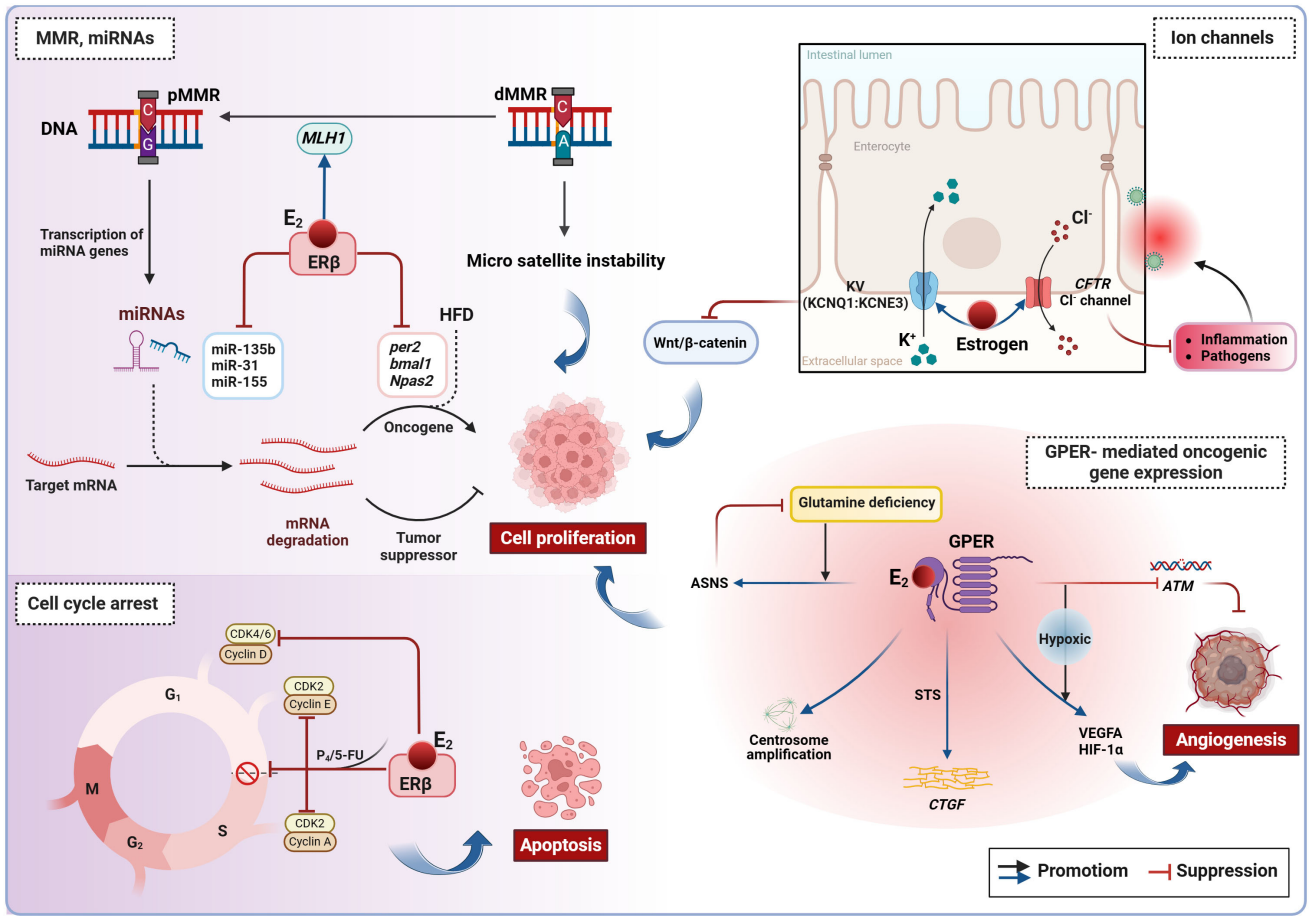
Estrogen-induced genetic and epigenetic alterations in CRC. Estradiol inhibits CRC cell proliferation by promoting DNA mismatch repair, regulating miRNAs, blocking the cell cycle, and modulating ion channels. However, in conditions of ERβ deficiency and hypoxia, estradiol can activate non-genomic signaling *via* GPER, thereby promoting oncogene expression. ASNS, asparagine synthase. ATM, Ataxia telangiectasia mutated; CRC, colorectal cancer; CTGF, connective tissue growth factor; E_2_, estradiol/17β-estradiol; ERβ, estrogen receptor beta; GPER, G protein-coupled estrogen receptor; HFD, high-fat diet; MMR, mismatch repair; dMMR, deficient MMR; pMMR, proficient MMR; P_4_, progesterone; STS, steroid sulfatase.

ERα and ERβ are classical nuclear transcription factors characterized by six domains, which include a DNA-binding domain and a ligand-binding domain (Acconcia et al., 2017; Khan et al., 2022). Upon activation by estrogen, the ligand, both ERα and ERβ undergo conformational changes, homodimerize, and subsequently bind to the estrogen response element located in the promoter regions of target genes. This binding modulates gene transcription, ultimately influencing cell growth, differentiation, and apoptosis (Mauvais-Jarvis et al., 2013). Generally, estrogen exerts its effects on CRC primarily through ERβ-mediated genomic signaling. Certain CRC types show mutation accumulation due to mismatch repair (MMR) system failure, notably loss of *MLH1* function (Lizardo et al., 2020). Estrogen has been found to inhibit MMR-proficient CRC while promoting MMR-deficient tumors (Honma et al., 2023). Estradiol enhances *MLH1* expression in CRC cells, heightens sensitivity to 5-FU, and synergistically inhibits tumor growth (Jin et al., 2010; Lu et al., 2017). It also suppresses certain miRNAs (miR-135b, miR-31, and miR-155) expression and upregulates *hMLH1* expression in CRC tissues or cells, regulating cancer cell differentiation, proliferation, and apoptosis (He et al., 2012). The impact of estradiol on miRNAs in various CRC cell lines seems to be associated with the expression level of ERβ (He et al., 2012). The circadian system affects cancer progression differently in males and females (Zhou et al., 2022; Levi et al., 2024). miR-34a, a CRC suppressor, notably inhibits the expression of the clock genes *per2* and *Bmal1*. While estradiol significantly reduces the proliferative and migratory activity of DLD1 cells, it does not have a substantial effect on miR-34a levels (Moravcik et al., 2023). However, estradiol seems to hinder CRC growth by modulating high-fat diet (HFD)-induced changes in the clock genes *Bmal1* and *Npas2 via* ERβ (Hases et al., 2020). Furthermore, when combined with progesterone or 5-FU, estradiol promotes ERβ expression in CRC cells, reduces the expression of the cell cycle markers CCND1/3, ultimately leading to cell cycle arrest in the sub-G1 phase and apoptosis (Mahbub, 2022; Mahbub et al., 2022). Changes in ion transporter activity are one of the epigenetic alterations identified in CRC. Estrogens are recognized as regulators of epithelial ion channels and influence the function of the voltage-gated K^+^ channel (KCNQ1:KCNE3) and the *CFTR* Cl^-^ channel (Anderson et al., 2019; Abancens et al., 2020). Both *KCNQ1* and *CFTR* are downregulated in CRC (Anderson et al., 2019; Abancens et al., 2020). KCNQ1 inhibits the Wnt/β-catenin pathway, thereby suppressing CRC cell proliferation and metastasis (Abancens et al., 2020). The absence of *CFTR* results in mucus blockage, inflammation, and microbial dysbiosis (Anderson et al., 2019). These findings suggest that estradiol directly inhibits CRC cell proliferation and induces apoptosis primarily through mechanisms such as promoting DNA mismatch repair, modulating miRNA and clock genes, arresting the cell cycle, and regulating ion channels (**Figure 2**). It is important to note that further validation of these results is necessary in future studies, considering variations in study methodologies, types of CRC cell lines, and concentrations and exposure times of estradiol.

##### 3.1.1.2 GPER- mediated oncogenic gene expression

In the absence of ERβ, estradiol initiates rapid signaling events primarily through the membrane receptor GPER, which contributes to pro-tumor effects in CRC (**Figure 2**). Typically, GPER signaling occurs via transactivation of the epidermal growth factor receptor. In addition to these rapid signaling events, GPER also regulates the *FOS* gene by activating signaling pathways involving cAMP, PI3K, and ERK, which promotes the formation of the transcription factor AP-1 and indirectly influences transcriptional activity (Arterburn and Prossnitz, 2023). As CRC advances, ERβ levels decrease while GPER remains expressed, making it a key player in estradiol’s impact on CRC. Clinical evidence indicates that high GPER expression is linked to a worse prognosis only in female patients with advanced CRC, not affecting survival in early-stage female patients or male patients at any stage (Bustos et al., 2017; Gilligan et al., 2017; Abancens et al., 2022; Lu et al., 2023). Under normoxic levels, estradiol inhibits the proliferation and migration of ERβ-negative CRC cells. However, under hypoxic conditions, GPER activated by estradiol promotes angiogenesis by suppressing the Ataxia telangiectasia mutated (*ATM*, a key MMR gene), and increasing VEGFA and HIF-1α expression (Bustos et al., 2017). Given the hypoxic nature of advanced CRC and the functional similarities between estrogen and hypoxia, this combined effect may explain the sex differences observed in female patients with advanced CRC and their lower survival rates when GPER expression is high. Furthermore, estradiol-induced GPER activation triggers centrosome amplification in HCT-116 and HT-29 cells, leading to the formation of alignment defects and lagging chromosomes that drive malignant progression (Buhler et al., 2023). Although estradiol does not affect the survival of *KRAS*-mutant CRC cells, these cells can adapt to nutrient scarcity by upregulating GPER and asparagine synthetase (ASNS) expression in the absence of glutamine (Lu et al., 2023). Previously mentioned as a bacterial enzyme facilitating the desulfuration of E_1_S and subsequent estradiol production (Thomas and Potter, 2013), STS stimulation leads to further GPER activation by estradiol, promoting connective tissue growth factor (CTGF*)* expression and stimulating CRC cell proliferation (Gilligan et al., 2017). Therefore, elevated STS activity is regarded as an indicator of poor prognosis in CRC. These results indicate that GPER, *ATM*, ASNS, and STS could serve as possible targets for therapy in CRC. However, it is also suggested that estrogen replacement therapy, tamoxifen and fulvestrant (known GPER agonists) commonly used in clinical practice, may have a negative impact on CRC prevention and treatment. Therefore, caution is advised for advanced CRC patients considering the use of these medications.

#### 3.1.2 Androgen

**Figure 3:**
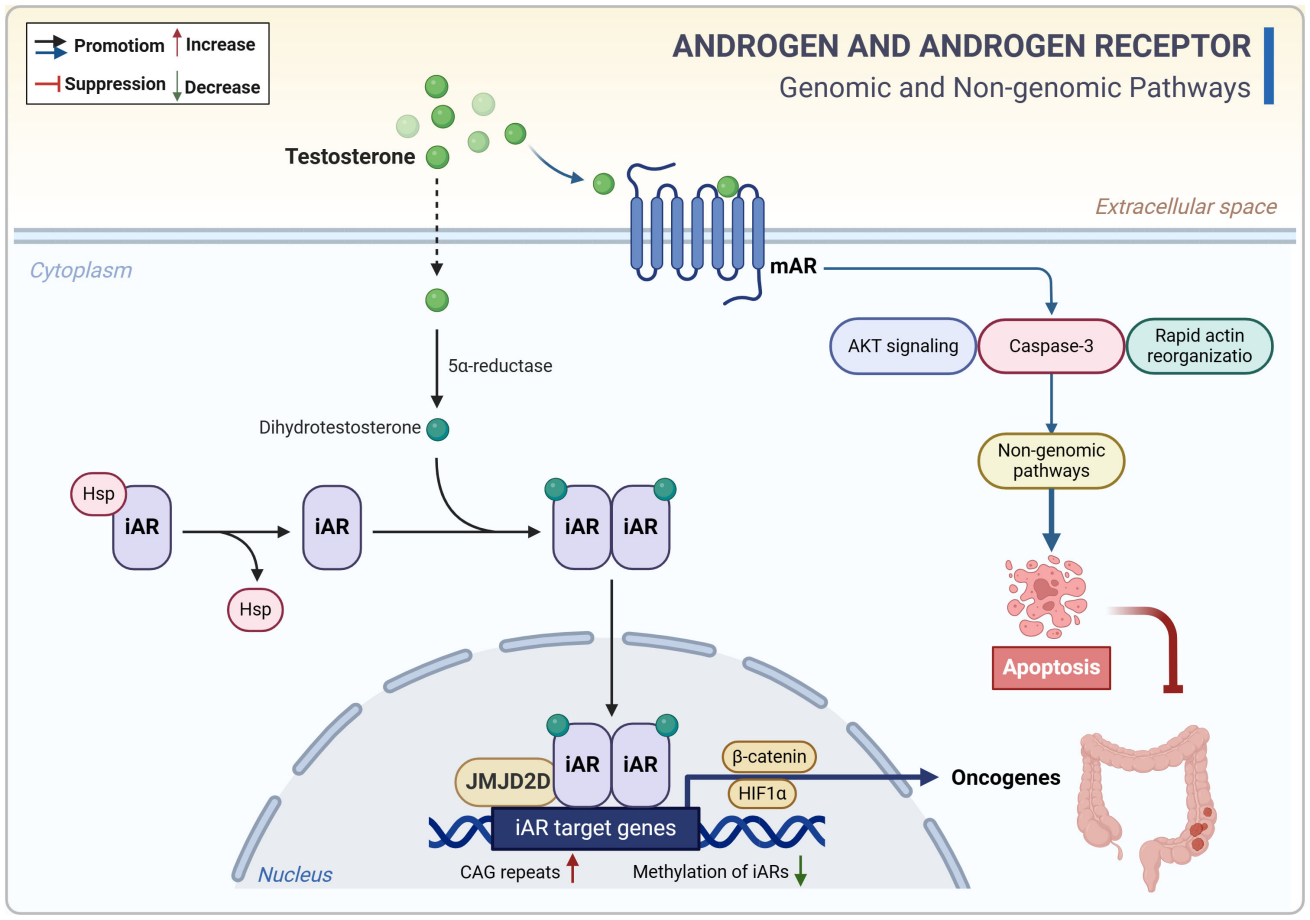
Androgen-related genomic and non-genomic signals in CRC. Androgens (testosterone) contribute to epigenetic dysregulation and CRC progression primarily through the nuclear receptors, whereas their interaction with membrane receptors may trigger apoptosis. CAG, cytosine-adenine-guanine. CRC, colorectal cancer; iAR, intracellular androgen receptor; mAR, membrane androgen receptor.

Like estrogens, androgens function through specific receptors known as androgen receptors (ARs). Structurally, ARs consist of two distinct subtypes: AR-α and AR-β. These isoforms act as ligand-activated transcription factors and exhibit differential expression in colon cancer tissue compared to healthy colon mucosa. At the mRNA level, ARs are present in both normal and colon cancer mucosa; however, at the protein level, both isoforms are expressed in normal colon mucosa. In contrast, AR-α is exclusively expressed in colon cancer, while the expression of AR-β is absent in colon cancer (Catalano et al., 2000). This indicates that the expression of AR-α in colon cancer cells may serve as a distinctive feature of CRC. Evidence suggests that testosterone may contribute to the development of CRC through multiple pathways, and serum free testosterone levels, together with carcinoembryonic antigen, are expected to serve as a potential biomarker for CRC (Roshan et al., 2016). Similar to estrogens, androgens act synergistically through intracellular ARs (iARs). iARs are DNA-binding transcription factors encoded by genes located on chromosome Xq11-12. In the general population, iARs contain varying numbers of cytosine-adenine-guanine (CAG) repeats that regulate gene expression and protein synthesis (Roshan et al., 2016). Patients with CRC tend to have longer CAG repeat lengths, increasing the risk of CRC in both males and females (Roshan et al., 2016). Epigenetic dysregulation is a susceptibility factor for tumorigenesis, with methylation of iARs genes potentially playing a role in sex differences in CRC (Xia et al., 2019; Chen et al., 2022b). An analysis of a cohort study indicated that hypomethylation of iARs in peripheral blood was associated with a higher risk of CRC, suggesting that iARs in peripheral blood could be a biomarker for CRC (Xia et al., 2019). The histone demethylase JMJD2D is an epigenetic factor that coordinates the activation of iARs. JMJD2D is highly expressed in the testis and promotes CRC progression by activating β-catenin and HIF1α to induce the expression of various oncogenes and glycolysis-related genes (Chen et al., 2022b). These findings imply that epigenetic dysregulation mediated by iARs could be a potential mechanism for testosterone-induced CRC development (**Figure 3**). However, there are conflicting views as well. Previous reports have shown that testosterone induces apoptosis in CRC through membrane ARs (mARs) (Gu et al., 2009, 2010, 2013). Activation of mARs, a class of G-protein-coupled receptors located on the cell surface, primarily occurs in CRC rather than in normal colonic mucosa (Roshan et al., 2016). Testosterone-albumin couplings selectively bind to mARs, inhibiting CRC cell invasion and migration while inducing apoptosis through the activation of Akt, caspase-3, and rapid actin reorganization (Gu et al., 2009, 2010, 2013). The discovery that androgens promote apoptosis in CRC cells aligns with certain clinical data suggesting that testosterone may have a potentially protective effect against CRC (Dashti et al., 2020). Overall, androgens contribute to epigenetic dysregulation and CRC progression primarily through the nuclear receptors, whereas their interaction with membrane receptors may trigger apoptosis (**Figure 3**). However, there is limited evidence regarding the direct impact of androgens on CRC genetics and epigenetics, necessitating further studies to validate these concepts.

### 3.2 Microecological dysregulation-induced genotoxicity

The intestinal microbiota is categorized into probiotic (e.g., *Bifidobacterium* and *Lactobacillus*), conditionally pathogens (e.g., *Enterobacter* and *Enterococcus*), and pathogens (e.g., *Salmonella*, *Proteus*, and *Escherichia coli*) (Chen et al., 2022a). CRC is linked to a reduction in overall gut microbial diversity and probiotics, along with an increase in pathogens compared to healthy individuals (Geng et al., 2013; Brennan and Garrett, 2016). The predominant flora in CRC includes bacteria like *Fusobacterium nucleatum*, *E. coli*, *Streptococcus gallolyticus*, and *Bacteroides fragilis*, particularly *F. nucleatum* (Lin et al., 2019; Koyande et al., 2022). *F. nucleatum* has been found in high levels in tumor tissues with microsatellite instability-high and CpG island methylator phenotype-high (Lin et al., 2019). In addition, *F. nucleatum* promotes the malignant phenotype of CRC cells by producing FadA adhesin that interacts with E-cadherin in epithelial cells and triggers the expression of inflammatory cytokines, transcription factors and Wnt genes (Castellarin et al., 2012; Fan et al., 2021; Wu et al., 2022). As mentioned previously, sex hormone signaling can influence the composition and diversity of gut microbiota in healthy individuals. Recent research suggests that CRC may also exhibit sex-specific differences in gut flora composition (Koyande et al., 2022; Yang et al., 2023). Clinical samples from CRC patients revealed that male patients had higher levels of *Bacteroides*, *Eubacterium*, and *Faecalibacterium*, while female patients had higher levels of *Bacteroides*, *Subdoligranulum*, and *Eubacterium*. Additionally, *Dorea* and *Bacteroides* were identified as key bacteria associated with sex and CRC, while *Blautia*, *Barnesiella* and *Anaerostipes* were among the most sexually diverse bacteria in CRC (Yang et al., 2023). This indicates that males and females may possess distinct gut microbiota that either promote or inhibit CRC progression. However, it remains unclear whether these microbial imbalances contribute to CRC development or are a result of the disease. Further investigation is needed to explore the relationship between sex-related bacteria, sex hormones, and dietary habits in the context of CRC.

**Figure 4:**
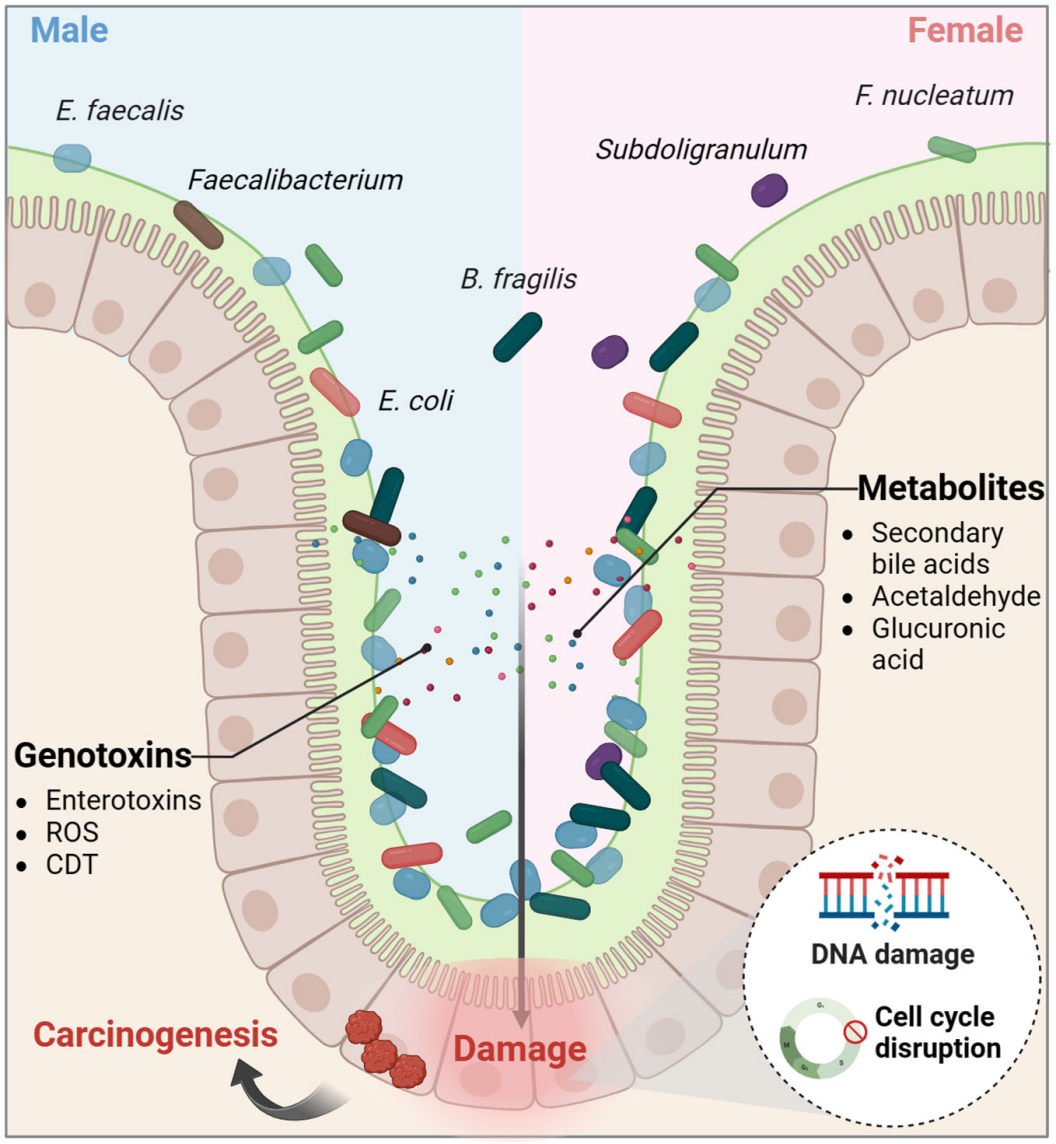
The intestinal microecological dysregulation-induced genotoxicity. Dysregulation of the intestinal microecology can lead certain microorganisms to produce virulence factors and metabolites that promote the development of colorectal cancer. CDT, cytolethal distending toxin; ROS, reactive oxygen species.

Microbial-induced genetic alterations that activate oncogenes or inactivate tumor suppressor genes play a significant role in colorectal carcinogenesis. Normally, gut microbes help protect the intestinal mucosa and prevent pathogen invasion by preventing DNA damage and maintaining the integrity of the intestinal barrier (Chen et al., 2022a). Dysregulation of the intestinal microecology can lead certain microorganisms to produce virulence factors and metabolites that promote the development of CRC (**Figure 4**). For example, some intestinal flora generate genotoxic toxins like enterotoxins and reactive oxygen species (ROS) from *B. fragilis* and *Enterococcus faecalis*, as well as the cytolethal distending toxin (CDT) produced by *E. coli*, which can cause DNA damage, disrupt the cell cycle, induce inflammation, and directly harm the intestinal epithelial cells (Lin et al., 2019; Chen et al., 2022a; Koyande et al., 2022; Qu et al., 2023; Wong and Yu, 2023). Additionally, bacterial metabolites such as secondary bile acids, acetaldehyde, and glucuronic acid, serve as energy sources for microbes and can accelerate CRC development by causing DNA damage, gene mutations, and the formation of oxygen radicals (Lin et al., 2019; Avuthu and Guda, 2022; Koyande et al., 2022). The genetic and epigenetic alterations triggered by these microorganisms are potential contributing factors to CRC, resulting in abnormal cell proliferation and malignant transformation of intestinal epithelial cells (Raskov et al., 2017). While there is some evidence indicating a possible causal link between gut microbiota and CRC, it remains unclear whether these bacteria act independently or in conjunction with other microorganisms to induce and advance colorectal carcinogenesis.

Taken together, both sex hormones and gut microbiota have dual effects on CRC. Estradiol inhibits CRC cell proliferation by mechanisms such as promoting DNA mismatch repair, regulating miRNAs and clock genes, blocking the cell cycle, and modulating ion channels. However, in conditions of ERβ deficiency and hypoxia, estradiol can activate non-genomic signaling through the membrane receptor GPER, thereby promoting oncogene expression. On the other hand, androgens contribute to epigenetic dysregulation and CRC progression primarily *via* nuclear receptor activation, while their interaction with membrane receptors can trigger apoptosis. Dysregulation of the intestinal microecology can result in the production of genotoxins and oncogenic metabolites, causing direct damage to colon cell DNA and promoting cancer development (**Figures 2**–**4**; **Box 2**). Despite indications of a potential direct causal link between sex hormone signaling, gut microbiome, and CRC, there is insufficient evidence to support the notion that the interplay between sex hormone signaling and the intestinal microbiota significantly contributes to sex-based differences in CRC. Current research predominantly focuses on exploring the impact of the sex hormone-gut microbiome axis on the colon tumor microenvironment (TME), particularly in terms of inflammation and immunity, as elaborated in the subsequent section.

Box 2 **Direct effects of sex hormones and the intestinal microbiota on CRC: genetic and epigenetic alterations.**

**Table:** 

**Estrogen** **Mismatch repair, miRNAs, cell cycle and ion channels** • Estradiol inhibits CRC growth by enhancing *MLHI* expression and DNA mismatch repair. • Estradiol inhibits miR-135b, miR-31, and miR-155 expression and regulates CRC cell differentiation and proliferation. • Estradiol inhibits CRC progression by regulating the clock genes *per 2,Bmall* and *Npas2* expression. • Estradiol in combination with progesterone or 5-FU decreases cell cycle markers expression and induces cell cycle arrest in sub-G1 phase. • Estrogen inhibits CRC cell proliferation and metastasis by regulating *KCNQl* K+ channel and *CFTR* Cl·channel.	**Androgen** • Androgens contribute to epigenetic dysregulation and CRC progression primarily through the nuclear receptors, whereas their interaction with membrane receptors may trigger apoptosis. • iARs contain varying amounts of CAG repeats, and CRC patients tend to have longer CAG repeats . • Methylation of iARs genes po·tentially playing a role in sex differences in CRC. • Testosterone-albumin couplings selectively bind to mARs, inhibiting CRC cell invasion and migration while inducing apoptosis through the activation of Akt, caspase-3, and rapid actin reorganization.
**GPER-mediated oncogenic gene expression** • High GPER expression is linked to a worse prognosis only in female patients with advanced CRC. • Under hypoxia and glutamine deficiency, estradiol-activated GPER inhibits *ATM* expression, and upregulates the levels of VEGFA, HIF-lα and ASNS, thereby promoting angiogenesis and metabolic reprogramming . • Estradiol-activated GPER triggers centrosome amplification and CTGF expression in CRC cells, leading to the formation of lagging chromosomes and CRC cell proliferation. • Estrogen replacement therapy, tamoxifen and fulvestrant may have a negative impact on CRC prevention and treatment.	**Microecological dysregulation-induced genotoxicity** • Dysregulation of the intestinal microecology can result in the production of genotoxins and oncogenic metabolites. • Enterotoxins fragilisin and ROS produced by *B.fragili s* and *E. faecali s*, as well as CDT prnduced by *E. coli*, can cause DNA damage and cell cycle d sruption in colon cells.Bacterial metabolites such as secondary bile acids, acetaldehyde, and glucuronic .acid, serve as energy sources for microbes and can accelerate CRC development by causing DNA damage, gene mutations, and the formation of oxygen radicals.

ASNS, asparagine synthetase; ATM, Ataxia telangiectasia mutated; CAG, cytosine-adenine-guanine; CDT, cytolethal distending toxin; CRC, colorectal cancer; GPER, G protein-coupled estrogen receptor; iARs, intracellular androgen receptors; mARs, membrane androgen receptors; ROS, reactive oxygen species.

## 4 Indirect pathways of sex hormones and the intestinal microbiota on CRC: tumor microenvironment

### 4.1 Inflammatory networks

**Figure 5:**
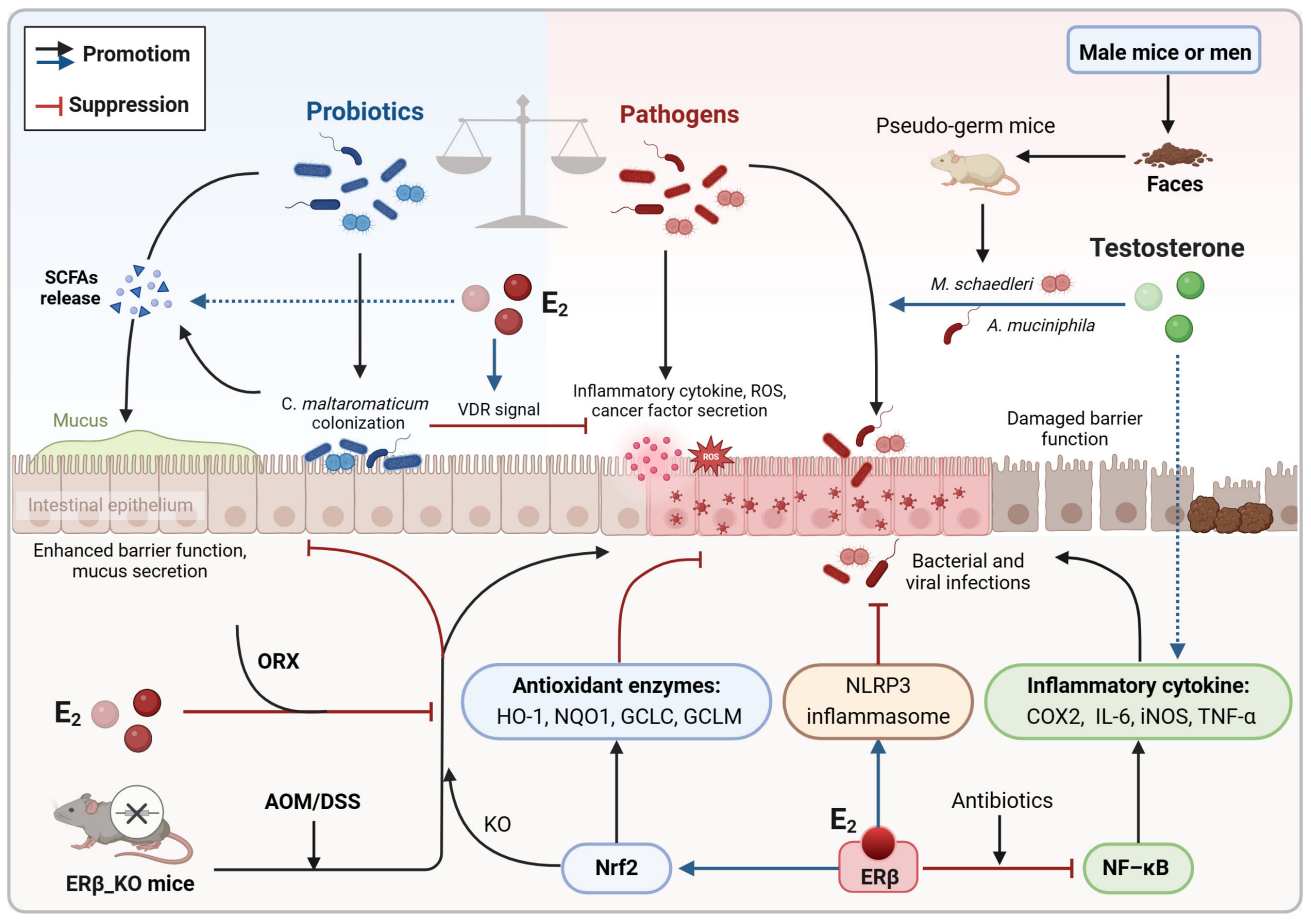
Co-regulation of colorectal inflammatory microenvironment by sex hormones and gut microbiota. Estrogen may slow the progression of CAC by promoting a more favorable gut microecology that reduces intestinal inflammation and inhibits oxidative stress damage. This includes increasing the diversity of the gut microbiome and the abundance of probiotics while simultaneously decreasing the abundance of pathogens. On the contrary, androgen may play a role in disrupting gut microecological balance, which could exacerbate colonic inflammation and heighten the susceptibility to intestinal tumor development. AOM/DSS, azoxymethane/dextran sodium sulfate; CAC, colitis-associated cancer; E_2_, estradiol/17β-estradiol; ERβ, estrogen receptor-beta; KO, knockout; ORX, orchiectomized; ROS, reactive oxygen species; SCFAs, short chain fatty acids; VDR, vitamin D receptor.

Inhibition of inflammatory signaling and pro-inflammatory cytokines expression is a key mechanism through which estrogen reduces the risk of colitis-associated cancer (CAC) (Son et al., 2019; Song et al., 2019). Previous studies have shown that ovariectomy worsens colonic inflammation and tumorigenesis in AOM/DSS model mice, while supplementation with 17β-estradiol (E2) significantly decreases inflammation and malignant progression. Mechanistically, estradiol binds to ERβ and inhibits the release of inflammatory mediators like COX-2, TNF-α, IFN-γ, and IL-6 by blocking NF-κB signaling (Armstrong et al., 2017; Son et al., 2019; Song et al., 2019; Son et al., 2020). On the other hand, estradiol enhances NLRP3 inflammasome expression, which helps reduce intestinal inflammation (Son et al., 2019). Chronic colitis can disrupt gut microbial balance and trigger colon carcinogenesis, which in turn leads to a further reduction in the gut microbiota diversity, especially in the absence of ERβ (Armstrong et al., 2017; Ibrahim et al., 2019; Lee et al., 2019; Song et al., 2020b). In the AOM/DSS model, modulating the gut microbiota with antibiotics lowered pro-inflammatory cytokines levels and colon tumorigenesis, with estradiol supplementation yielding similar results (Lee et al., 2019; Song et al., 2020b). Estradiol increased gut microbial diversity, boosted commensal bacteria levels, and reduced opportunistic pathogens and the F/B ratio in male and female mice (Song et al., 2020b). Probiotics stimulate anti-inflammatory cytokines production, while pathogens trigger the release of inflammatory mediators, pro-inflammatory toxins, and ROS (Lin et al., 2019; Fan et al., 2021; Koyande et al., 2022). In the absence of ERβ, both male and female AOM/DSS mice exhibited decreased gut microbial diversity, particularly male mice with an abundance of flora linked to cell motility and metabolism (Ibrahim et al., 2019). *Carnobacterium maltaromaticum* has shown significant inhibition of intestinal tumor formation in female mice but has minimal impact on male mice, indicating its potential anticancer activity in the presence of estrogen (Li et al., 2023). Estradiol is thought to enhance the attachment and colonization of *C. maltaromaticum* by upregulating colonic SLC3A2 expression. The colonization of *C. maltaromaticum* leads to a shift in gut microbiota composition, increasing beneficial butyric acid-producing bacteria like *Faecalibacterium prausnitzii* and *Lachnispiraceae bacterium* while reducing levels of opportunistic pathogens such as *Bacteroides vulgatus* and *Muribaculum intestinale*. These beneficial bacteria activate vitamin D receptor (VDR) signaling, reduce mucosal inflammation, and maintain intestinal barrier function (Li et al., 2023). These findings suggest that estrogen and ERβ contribute to a favorable gut microbiota, crucial for mitigating inflammatory responses and preventing colorectal carcinogenesis (**Figure 5**). Modulating the gut microbiota using antibiotics or estrogens may hold promise as a potential therapeutic approach for CAC. It is important to note that the AOM/DSS model primarily simulates CRC associated with inflammatory bowel disease. In addition to its connections to sex hormones and an imbalance in intestinal flora, the malignant progression of this cancer is also linked to a reduced rate of epithelial cell renewal and apoptosis. Furthermore, this progression is associated with a decrease exposure time to genetic mutants (Girardi et al., 2018).

Oxidative stress can lead to an increase in intracellular oxygen free radicals and ROS, which in turn can activate inflammatory responses. During the initial phases of colitis, estradiol boosts the expression of Nrf2 and its associated antioxidant enzymes HO-1, NQO1, GCLC, and GCLM, thereby reducing oxidative stress damage and inflammatory reactions (Son et al., 2019; Song et al., 2019). Nonetheless, in the absence of Nrf2, estradiol may hinder the progression of distal CRC through an ERβ-dependent pathway (Song et al., 2020a). Apart from upregulating antioxidant enzymes, estradiol also triggers the expression of neuroglobin, an oxidative stress sensor. In the absence of oxidative stress, estradiol induces apoptosis in CRC cells; however, in the presence of high oxidative stress, neuroglobin interacts with cytochrome *C* to inhibit the subsequent apoptotic process, thereby counteracting the pro-apoptotic impact of estradiol (Fiocchetti et al., 2015). Emerging evidence indicates that the Nrf2 genotype can influence the intestinal microbiota composition (Song et al., 2021b). *B. vulgatus* promotes inflammatory responses and the malignant advancement of colon adenomas in cases where gut barrier function is compromised. Treatment with AOM/DSS leads to an increase in the abundance of *B. vulgatus* in both male and female mice. Conversely, Nrf2 knockout results in a decrease in the abundance of the probiotic *Lactobacillus murinus* in male mice. Furthermore, the levels of the intestinal pathogen *Akkermansia muciniophila* are elevated in male mice irrespective of Nrf2 knockout (Song et al., 2021b). These findings suggest that estrogen may delay the progression of CAC by fostering a more favorable gut microbiota that helps mitigate oxidative stress damage and intestinal inflammation (**Figure 5**).

In comparison to estrogen signaling, androgen seems to contribute to dysregulation of the intestinal microecology, colonic inflammation, and tumor progression (Song et al., 2021a, 2023b; Wang et al., 2023). Studies using the AOM/DSS model have shown that orchiectomy in male mice significantly reduced the severity of colitis and distal colonic adenomas (Song et al., 2021a, 2023b). Additionally, mice without testes had higher gut microbial diversity and increased F/B ratios (Song et al., 2023b). When testosterone was supplemented, it led to the upregulation of inflammatory mediators like COX-2 and iNOS, as well as an increase in opportunistic pathogens such as *Mucispirillum schaedleri* or *Akkermansia muciniphila*, exacerbating colonic inflammation and submucosal invasive carcinoma (Song et al., 2021a, 2023b). Moreover, in pseudo-germ mice that received fecal samples from male mice or men, *A. muciniphila* was significantly enriched while the probiotic *Parabacteroides goldsteinii* was reduced, resulting in increased intestinal inflammation and disruption of barrier function (Wang et al., 2023). Mechanistically, the metabolites from these pathogens activate the glycerophospholipid metabolic pathway, ultimately worsening colon tumorigenesis in males (Wang et al., 2023). These findings suggest that androgen, particularly testosterone, may induce intestinal microecological dysregulation and chronic inflammation, potentially contributing to sex differences in CRC (**Figure 5**).

Overall, estrogens have been shown to potentially reduce intestinal inflammation and slow the progression of CAC by promoting a more favorable gut microecology. This includes increasing gut microbiome diversity and commensal bacteria abundance, while decreasing pathogen abundance. Conversely, androgen may contribute to intestinal microecological dysregulation, leading to worsened colonic inflammation and increased risk of intestinal tumor formation (**Figure 5**; **Box 3**). However, current evidence is limited, with most studies focusing on differences in strains influenced by sex hormones or sex, rather than exploring the specific molecular mechanisms underlying the interaction between sex hormones and the intestinal microbiota in relation to intestinal inflammation.

Box 3 **Indirect pathways of sex hormones and the intestinal microbiota on CRC: tumor microenvironment.**

**Table:** 

**Inflammatory networks** **Estrogen** • Estradiol attenuates intestinal inflammation by blocking NF-kB signaling and enhancing NLRP3 inflammasome expression. • Supplementation with estradiol or antibiotics to modulate the gut microbiota reduces pro-inflammatory cytokine levels and colon tumorigenesis . • Estradiol enhances gut colonization of C. *maltaromaticum*, which further promotes SCFAs-producing bacteria enrichment and activates vitamin D receptor signaling, thereby reducing mucosa! inflammation and maintaining gut barrier function. • Estrogen and ERβ slow the progression of CAC by promoting a more favorable intestinal microecology .	**Immune microenvironent** **Estrogen** • Estradiol reverses immunosuppressive TME by downregulating PD-L1 expression and modulating the population of infiltrating immune cells and tumor-associated cells. • Estradiol reduces immunosuppressive factor level in EVs and inhibits MC38 colon tumor growth. • Combined treatment with estradiol and αPD-L1 increase the abundance of intestinal probiotics *(P. goldsteinii* and *L. murinus) *while decreasing the presence of opportunistic pathogens *(Enterobacteriaceae* family).
**Androgen** • Orchiectomy in male mice significantly reduced the severity of colitis and distal colonic adenomas. • Testosterone supplementation upregulates the levels of inflammatory mediators as well as opportunistic pathogens (*M schaedleri* or *A. muciniphila*), exacerbating colonic inflammation and submucosal invasive carcinoma. • The intestines of pseudo-germ mice receiving fecal samples from male mice or men were significantly enriched in *A. muciniphila* and reduced in the probiotic *P. goldsteinii*, leading to increased intestinal inflammation. • Androgen seems to contribute to dysregulation of the gut microecology, colonic inflammation, and tumor progression.	**Androgen** • αPD-L1 treatment appears to be more effective in male patients than in female patients. • αPD-L1 reduces testosterone levels without affecting the gut microbiota in male mice, but decreases the abundance of *Lachnospiraceae* (associated with positive responses to ICIs) in females. • Reducing testosterone levels enhances the efficacy of immunotherapy. • Sex differences in gut microbiota need to be considered when using antibiotics to manage ICIs-associated colitis.
**Oxidative stress** • Estradiol boosts the expression of Nrf2 and its associated antioxidant enzymes, thereby reducing oxidative stress damage and inflammatory reactions. • Nrf2 knockout results in a decrease in the abundance of the probiotic *L. murinus* in AOM/DSS male mice. • In the presence of high oxidative stress, neuroglobin interacts with cytochrome *C* to inhibit the subsequent apoptotic process, thereby counteracting the pro-apoptotic impact of estradiol.	**The intestinal microbiota and host immune system** • Pathogens (e.g., *F. nucleatum*) can induce MDSCs, M2-like TAMs, and Th1 cells to infiltrate and form intestinal inflammation, triggering host immunosuppression or aberrant immune responses. •Certain gut probiotics can stimulate T cell production, activate NK cells, boost macrophage phagocytic activity, and enhance the body's anti-tumor immune response.

AOM/DSS, azoxymethane/dextran sodium sulfate; CAC, colitis-associated cancer; CRC, colorectal cancer; ERβ, estrogen receptor-beta; EVs, extracellular vesicles; ICIs, immune checkpoint inhibitors; MDSCs, myeloid-derived suppressor cells; PD-L1, programmed death-ligand 1; SCFAs, short chain fatty acids; TAMs, tumor-associated macrophages; TME, tumor microenvironment.

### 4.2 Immune microenvironment

**Figure 6:**
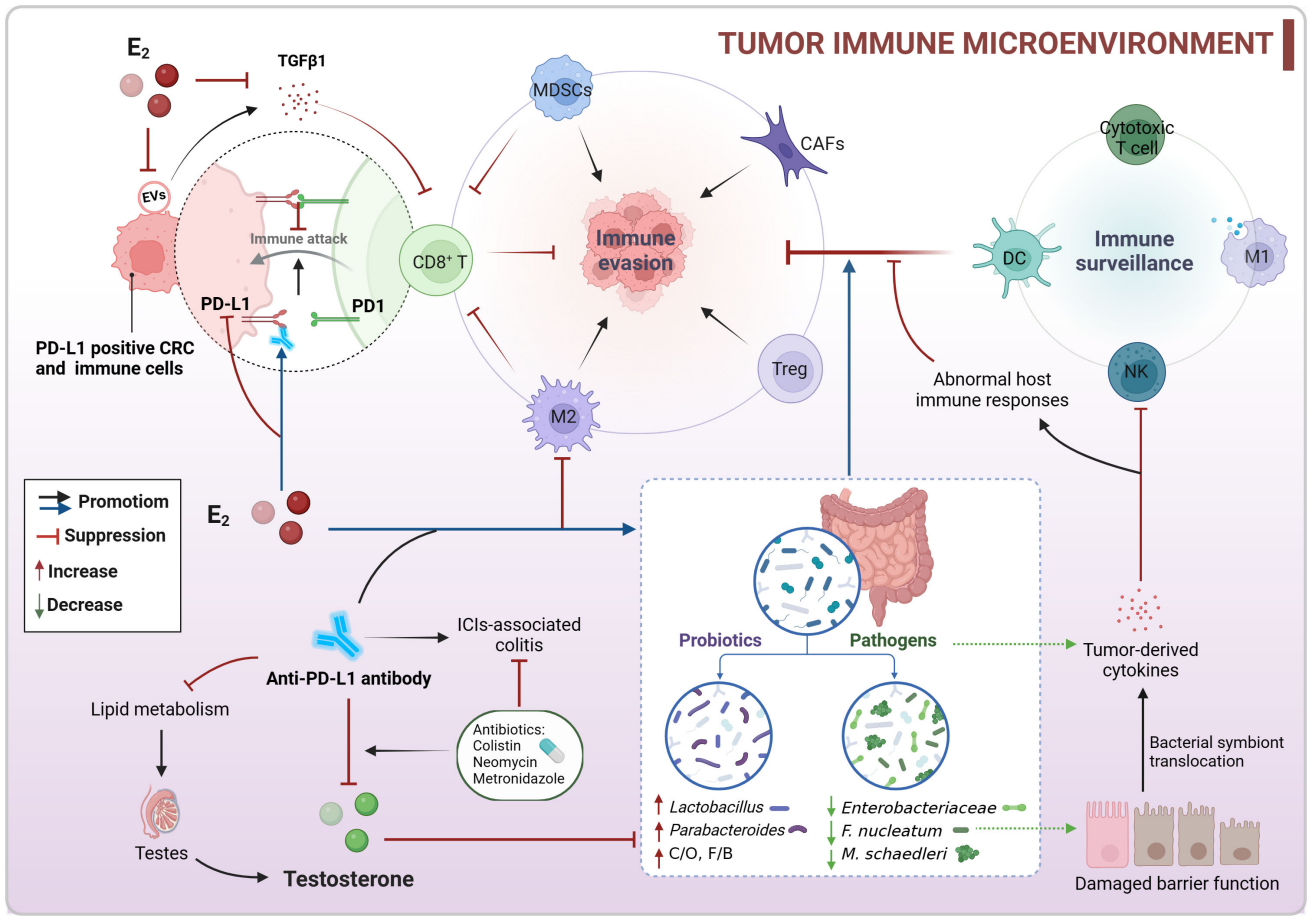
Co-regulation of colorectal immune microenvironment by sex hormones and gut microbiota. Estradiol may enhance the body’s anti-tumor immunity by downregulating PD-L1 expression, modulating the population of infiltrating immune cells, and fostering an intestinal microecology that supports immune recovery to counteract the suppressive TIME. Conversely, testosterone could have a negative impact on αPD-L1 therapy, and reducing testosterone levels might change gut microbial composition and enhance the effectiveness of immunotherapy. CRC, colorectal cancer; C/O, commensal bacteria/opportunistic pathogens; E_2_, estradiol/17β-estradiol; EVs, extracellular vesicles; F/B, *Firmicutes*/*Bacteroidetes*; ICIs, immune checkpoint inhibitors; MDSCs, myeloid-derived suppressor cells; PD-L1, programmed death-ligand 1; TIME, tumor immune microenvironment.

It is well known that the expression of programmed death-ligand 1 (PD-L1) in tumor cells or immune cells can inhibit T cell cytotoxicity (Dammeijer et al., 2020). In both AOM/DSS and MC38 colon tumor models, there was a high presence of PD-L1-positive tumor cells, M2-like tumor-associated macrophages (TAMs), myeloid-derived suppressor cells (MDSCs), Treg cells, and cancer-associated fibroblasts (CAFs), along with a low presence of cytotoxic CD8^+^ T cells (Jiang et al., 2021; Kang et al., 2021; Song et al., 2022). Estradiol supplementation was found to reverse this suppressive immune microenvironment and enhance the body’s anti-tumor immune response (Jiang et al., 2021; Kang et al., 2021; Song et al., 2022). Additionally, extracellular vesicles (EVs) derived from MC38 colon tumors were also found to contribute to the immunosuppressive TME. Estradiol was shown to decrease the levels of the immunosuppressive factor TGF-β1 in EVs, ultimately inhibiting MC38 tumor growth (Jiang et al., 2021). Interestingly, different concentrations of estradiol did not impact the viability of MC38 cells or the expression of PD-L1 in these cells [96]. Furthermore, estradiol treatment before injecting MC38 cells led to a significant reduction in tumor weight, whereas treatment after cell injection had no effect on tumor growth (Song et al., 2022). Based on the discovery that estradiol reduces PD-L1 expression in colon tumor tissues to impede tumor growth, researchers also observed that pre-treatment with estradiol before αPD-L1 therapy led to alterations in the gut microbiota composition and diversity in male mice (Song et al., 2020b, 2022, 2023a). The combination of estradiol and αPD-L1 treatment resulted in increased levels of the intestinal probiotic *Parabacteroides goldsteinii* and *Lactobacillus murinus* group, while reducing the presence of the opportunistic pathogen *Enterobacteriaceae* family in MC38 male mice (Song et al., 2023a). However, changes in the F/B ratios varied among different models, with estradiol treatment alone or in combination with αPD-L1 increasing F/B ratios in MC38 mice, but significantly decreasing in AOM/DSS mice (Song et al., 2020b, 2023a). Despite a decrease in the relative abundance of certain commensal bacteria (PAC000664 and *Phocea*) and opportunistic pathogens (*Pseudoflavonifractor* and *Neglecta*) in estradiol-treated AOM/DSS male mice, the ratio of commensal bacteria to opportunistic pathogens remained elevated (Song et al., 2020b). Overall, these findings suggest that estradiol downregulates PD-L1 expression, modulates the population of infiltrating immune cells and tumor-associated cells, and fosters a gut microecological environment conducive to immune recovery. Through its interaction with the intestinal microbiota, estradiol collaborates to reverse the suppressive immune microenvironment and enhance the body’s anti-tumor immunity, ultimately hindering the progression of CRC (**Figure 6**).

Estradiol enhances the efficacy of αPD-L1, but clinical data indicates that αPD-L1 treatment is more effective in male patients than in female patients (Wang et al., 2021). In the MC38 colon tumor model, it was observed that αPD-L1 reduced testosterone levels in male mice without affecting sex hormone levels in females. αPD-L1 did not impact the gut microbiota in males, but it decreased the abundance of *Lachnospiraceae* in females, a group associated with positive responses to immune checkpoint inhibitors (ICIs). Mechanistically, αPD-L1 disrupted lipid metabolism and caused inflammation in testes, leading to decreased testosterone production. Moreover, the administration of antibiotic colistin during anti-PD-L1 treatment further lowered testosterone levels and increased estradiol levels in male mice, resulting in alterations in gut microbiota composition and function (Wang et al., 2021). These findings suggest that reducing testosterone levels enhances the efficacy of immunotherapy (**Figure 6**). The use of specific narrow-spectrum antibiotics, such as neomycin and metronidazole, alleviated ICIs-associated colitis in MC38 mice and improved the anti-tumor efficacy of αPD-L1 in male mice, while negatively affecting its efficacy in female mice. These differences were linked to sex-specific variations in the intestinal microbiota, particularly in the abundance of *Muribaculaceae* and *Lachnospiraceae* (Jing et al., 2022). The study highlights the importance of considering sex differences in the gut microbiota when using antibiotics to manage ICIs-associated colitis.

The intestinal microbiota plays a crucial role in shaping the host immune system’s development and function, which is essential for maintaining the key features of the host-microbe symbiosis (Zheng et al., 2020). Whereas the immune system is capable of sex differences, the gut microbiota is simultaneously influenced by sex hormones that control sex differences (Rizzetto et al., 2018). There is extensive communication between the gut microbiota and host immunity, with potential implications for cancer immune surveillance. For instance, the presence of *F. nucleatum* in the colorectal TME can directly impede the killing of tumors by NK cells and is associated with lower CD3^+^ T cell levels (Gur et al., 2015; Mima et al., 2015). Pathogenic bacteria like *F. nucleatum*, *Klebsiella pneumoniae*, and *Peptostreptococcus anaerobius* can enhance the activity of tumor-derived cytokines, leading to the recruitment of MDSCs and M2-subtype TAMs, thereby supporting CRC progression (Wong and Yu, 2023). In mice lacking both *NOD2* and *CYBB*, the pathogen *Mucispirillum schaedleri* can induce Th1 cell-mediated intestinal inflammation (Caruso et al., 2019). Furthermore, inflammation-induced disruption of the gut barrier can result in the translocation of bacterial symbionts into the mucosal layer, triggering abnormal host immune responses and tissue damage (Zheng et al., 2020). In contrast, certain intestinal probiotics can stimulate T cell production, activate NK cells, boost macrophage phagocytic activity, and enhance the body’s anti-tumor immune response (Lin et al., 2019; Fong et al., 2020; Zheng et al., 2020; Koyande et al., 2022) (**Figure 6**). These discoveries highlight the significant role of the intestinal microbiota in cancer immune surveillance and immunotherapy, suggesting its potential for optimizing the immunotherapeutic response in CRC patients.

Collectively, estradiol may enhance the body’s anti-tumor immunity by downregulating PD-L1 expression, modulating the population of infiltrating immune cells, and fostering an intestinal microecology that supports immune recovery to counteract the suppressive tumor immune microenvironment (TIME). Conversely, testosterone could have a negative impact on αPD-L1 therapy, and reducing testosterone levels might change gut microbial composition and enhance the effectiveness of immunotherapy (**Figure 6**; **Box 3**). These results indicate that sex hormones and gut microbiome may be pivotal in CRC immunotherapy. Estradiol and specific probiotics could be valuable targets for boosting αPD-L1 efficacy. Moreover, sex-specific variations in the intestinal microbiota should be taken into account when using antibiotics to manage ICI-associated colitis.

## 5 The contribution of environmental factors to sex hormones and intestinal microbiota in CRC

Common research primarily focuses on the effects of endogenous sex hormones on intestinal microbiota and CRC. Notably, specific lifestyle choices and hormone therapies also influence human sex hormone levels and gut microbiota abundance. Previous studies have indicated that various dietary patterns affect estrogen levels differently in premenopausal women. Estrogen concentrations were found to be inversely associated with overall diet quality, as assessed by the Alternative Healthy Eating Index model, but not with diet quality evaluated by the Dietary Approaches to Stop Hypertension or the alternative Mediterranean Eating pattern (Hirko et al., 2016). Evidence suggests that low energy intake can adversely affect serum testosterone concentrations, and certain vitamins and minerals are crucial for testosterone synthesis (Zamir et al., 2021). Additionally, a high-cholesterol diet may lead to reduced serum testosterone levels in rats (Liu et al., 2023). However, some clinical trials have reported that micronutrients do not significantly impact free testosterone and estradiol levels (Janjuha et al., 2020). Additionally, long-term follow-up evidence from the Women’s Health Initiative does not support the addition of vegetables, fruits, and grains to a low-fat diet in menopausal women for the prevention of CRC (Manson et al., 2024). It is important to note that these findings are limited, and all experiments or trials exhibit notable methodological limitations. Phytoestrogens are present in a wide variety of foods and plants, and they can disrupt the endocrine system by interfering with the hypothalamic-pituitary-gonadal axis, which regulates estrogen secretion. The effects of phytoestrogens on the body can vary depending on the individual’s life stage. Additionally, phytoestrogens can alter levels of sex hormone-binding globulin and influence the biological activity of circulating estrogen and androgen (Domínguez-López et al., 2020). Studies have demonstrated that the phytoestrogen isoflavones stimulate the synthesis of hormone-binding globulins in hepatocellular carcinoma cells and inhibit aromatase and other enzymes involved in steroid hormone synthesis (Domínguez-López et al., 2020). Isoflavones can be converted to S-estrone by intestinal bacteria; however, not all individuals produce S-estrone. Results from trials involving individuals at high risk of CRC suggest that isoflavones may reduce insulin-growth factor, but this effect appears to occur only in those who produce estramustrol (Usui et al., 2013). In addition to the effects of dietary and exogenous hormones on sex hormone levels, hormone therapy significantly influences the onset of CRC. Research indicates that individuals undergoing postmenopausal hormone replacement therapy can reduce their risk of CRC by over 20% (Murphy et al., 2015). Clinical trials suggest that estrogen-progesterone therapy may decrease women’s absolute risk of CRC; however, the overall health risks associated with the use of estrogen plus progesterone, such as a potential increased risk of breast cancer, outweigh the benefits (Rossouw et al., 2002; Genazzani et al., 2021). While the evidence remains inconsistent, it appears that overall menopausal hormone therapy is safe for many cancer patients (Hickey et al., 2024).

The composition and diversity of the intestinal microbiota are influenced by host physiology and environmental factors. Increasing evidence suggests that environmental factors such as high-fat diet (HFD), smoking, alcohol consumption, overweight, and obesity can also impact CRC by inducing significant changes in the intestinal microbiota composition (O’Keefe, 2016; Capurso and Lahner, 2017; Haziman et al., 2019; Song and Chan, 2019; Zheng et al., 2020; Chang et al., 2024). Men, often influenced by social background factors, tend to consume more fat, smoking, and alcohol than women, contributing partially to the increased risk of CRC in men (Sun et al., 2023). Therefore, it is necessary to differentiate between the effects of dietary and lifestyle choices and true biological sexual dimorphism on CRC. A substantial body of evidence indicates that HFD can contribute to the progression of CRC by impacting the intestinal microbiota and its metabolites, as well as influencing immune cell function (Devkota et al., 2012; Cheng et al., 2016; O’Keefe, 2016; Yang et al., 2022; Song et al., 2023c; Cross et al., 2024). HFD has been shown to disrupt gut barrier function, leading to increased proliferation and metastasis of CRC cells by altering the balance of intestinal pathogens (such as *Alistipes* sp. *Marseille*-P5997) and harmful metabolites while reducing probiotics like *Parabobacterides distasonis* (Yang et al., 2022). Oophorectomy has been found to increase intestinal permeability and inflammation, promoting a microbiota composition associated with metabolic dysfunction, which is further exacerbated by HFD (Cross et al., 2024). Furthermore, HFD elevates levels of secondary bile acids and *Bilophila wadsworthia*, leading to Th1-type immune responses (Devkota et al., 2012), and decreases butyric acid levels, disrupting intestinal dendritic cell homeostasis (Cheng et al., 2016) and promoting colitis and CAC development. Additionally, HFD in conjunction with the *HNF1A* ^A98V^ variant activates β-catenin, contributing to colon polyp formation (Song et al., 2023c). Untimely dietary intake interacts with alcohol consumption to accelerate alcohol-associated CRC by decreasing the population of SCFAs-producing bacteria (Bishehsari et al., 2020). Dietary habits, such as high consumption of red and processed meats, low intake of dietary fiber, and alcohol consumption, are also recognized as risk factors for CRC. Preservatives in red meat, processed meats and dairy products produce chemicals during cooking. These chemicals are metabolized by intestinal bacteria to produce harmful metabolites that promote colonic inflammation and tumors (Viennois et al., 2017; Song et al., 2020c). Diets rich in fiber support a beneficial relationship between gut microbes and their hosts. Fiber fermentation generates SCFAs like butyrate, propionate, and acetate, which are crucial for the energy supply of gut microbiota and the maintenance of colonic mucosal health (O’Keefe, 2016). Exposure to alcohol and smoking, in addition to dietary habits, can result in notable alterations in the composition of the intestinal microbiota. This includes a reduction in ‘anti-inflammatory’ bacteria from the *Firmicutes* phylum, such as *Lactococcus*, *Pediococcus*, and *Ruminococcus* sp., and an increase in ‘pro-inflammatory’ bacteria like *Bacteroidetes* and *Verrucomicrobia*. These changes can disrupt mucosal barrier function, trigger inflammation, and promote bacterial translocation (Capurso and Lahner, 2017).

Taken together, the impact of dietary patterns and lifestyle on the composition and metabolism of the intestinal microbiota is significant. Environmental factors such as diet and lifestyle choices may mask differences in gut microbial composition related to sex hormone status. This makes it challenging to pinpoint the correlation between individual dietary habits, lifestyle choices, and sexual dimorphism in the intestinal microbiota. Moreover, these results suggest that high-sugar, high-fat, and low-fiber diets, along with lifestyle habits like smoking and alcohol consumption, are risk factors for CRC. These findings offer valuable insights for the prevention of this disease.

## 6 Conclusion and future perspectives

In conclusion, sex hormones shape the composition and diversity of the gut microbiota, which reciprocally influences sex hormone levels. This bidirectional communication is crucial for understanding sex disparities in CRC. Both sex hormones and the intestinal microbiota are directly important for CRC cells as they contribute to genetic and epigenetic alterations. Moreover, they indirectly influence CRC onset, progression, and therapeutic responses by modulating inflammation and the immune microenvironment. For instance, estrogen restrains CRC cell proliferation by enhancing DNA repair, miRNA regulation, and ion channel modulation. In the absence of ERβ, estradiol activates non-genomic signaling via GPER, augmenting oncogene expression. Conversely, androgens promote epigenetic dysregulation and CRC advancement through nuclear receptor activation while inducing apoptosis via membrane receptors. Dysbiosis of the gut microbiota can result in the production of genotoxins and oncogenic metabolites by certain microbes, directly damaging colonic cell DNA and promoting tumorigenesis. Regarding the TME, estrogen fosters a favorable gut microecology by enhancing microbiome diversity and commensal bacteria abundance while suppressing pathogen levels, thereby mitigating intestinal inflammation and reversing immunosuppression. Conversely, androgens may disrupt intestinal microecology, diminish αPD-L1 efficacy, and exacerbate colonic inflammation and tumor growth. It is noteworthy that diet and lifestyle choices significantly shape gut microbiome composition, potentially masking differences due to sex hormone status. These findings suggest that targeting sex hormone receptors could be a promising strategy for intervening in CRC, with the intestinal microbiota serving as a potential biomarker or prognostic indicator for CRC. Estradiol and specific probiotics may enhance αPD-L1 efficacy. Additionally, considering sex-related differences in the intestinal microbiota when using antibiotics to manage ICIs-associated colitis is crucial. Overall, these insights offer valuable guidance for leveraging hormone replacement therapy, immunotherapy, and fecal microbiota transplantation in CRC management.

However, the direct contribution of interactions between sex hormone signaling and the intestinal microbiota to sex differences in CRC lacks sufficient evidence. Existing studies often focus on strain differences related to sex hormones or sex, without elucidating the specific molecular mechanisms through which these interactions lead to inflammation and immunosuppression. Future research should prioritize investigating how the interplay between sex hormones and the intestinal microbiota affects CRC genetics and epigenetics and delve into the mechanisms by which they influence the tumor microenvironment. Epidemiological evidence on the impact of the intestinal microbiota on CRC risk in men and women primarily derives from retrospective studies on patients already diagnosed with CRC. It remains unclear whether the observed gut microbial changes are a cause or a consequence of CRC development. Prospective studies that collect detailed gut microbiome data before individuals develop CRC are essential for better understanding the long-term effects of environmental factors (i.e., diet and lifestyle) on the intestinal microbiota and their implications for CRC prevention in both sexes. Human data on the role of gut microbiome alterations in CRC treatment are limited. The effects of the intestinal microbiota on laboratory mice may differ significantly from those in humans, with mice possessing a natural wild microbiota being more resilient to environmental challenges and responding to immunotherapy in a manner closer to humans. Overall, to develop sex hormone- and gut microbiome-based therapies, a deeper comprehension of the complex interactions between sex hormones, the intestinal microbiota, and sexual dimorphism in CRC is necessary. The successful translation of these therapies into clinical practice urges standardized, rigorous preclinical and clinical intervention investigations.
